# Colon Cancer Disease Diagnosis Based on Convolutional Neural Network and Fishier Mantis Optimizer

**DOI:** 10.3390/diagnostics14131417

**Published:** 2024-07-02

**Authors:** Amna Ali A. Mohamed, Aybaba Hançerlioğullari, Javad Rahebi, Rezvan Rezaeizadeh, Jose Manuel Lopez-Guede

**Affiliations:** 1 Department of Material Science and Engineering, University of Kastamonu, Kastamonu 37150, Turkey; amnaalkmati@gmail.com; 2 Department of Physics, University of Kastamonu, Kastamonu 37150, Turkey; aybaba@kastamonu.edu.tr; 3 Department of Software Engineering, Istanbul Topkapi University, Istanbul 34087, Turkey; 4 Department of Physics, Faculty of Science, University of Guilan, Rasht P.O. Box 41335-1914, Guilan, Iran; r.rezaeizade@gmail.com; 5 Department of Systems and Automatic Control, Faculty of Engineering of Vitoria-Gasteiz, University of the Basque Country (UPV/EHU), C/Nieves Cano 12, 01006 Vitoria-Gasteiz, Spain

**Keywords:** convolutional neural network, metaheuristic methods, FMO, Fishier Mantis Optimizer, colon cancer

## Abstract

Colon cancer is a prevalent and potentially fatal disease that demands early and accurate diagnosis for effective treatment. Traditional diagnostic approaches for colon cancer often face limitations in accuracy and efficiency, leading to challenges in early detection and treatment. In response to these challenges, this paper introduces an innovative method that leverages artificial intelligence, specifically convolutional neural network (CNN) and Fishier Mantis Optimizer, for the automated detection of colon cancer. The utilization of deep learning techniques, specifically CNN, enables the extraction of intricate features from medical imaging data, providing a robust and efficient diagnostic model. Additionally, the Fishier Mantis Optimizer, a bio-inspired optimization algorithm inspired by the hunting behavior of the mantis shrimp, is employed to fine-tune the parameters of the CNN, enhancing its convergence speed and performance. This hybrid approach aims to address the limitations of traditional diagnostic methods by leveraging the strengths of both deep learning and nature-inspired optimization to enhance the accuracy and effectiveness of colon cancer diagnosis. The proposed method was evaluated on a comprehensive dataset comprising colon cancer images, and the results demonstrate its superiority over traditional diagnostic approaches. The CNN–Fishier Mantis Optimizer model exhibited high sensitivity, specificity, and overall accuracy in distinguishing between cancer and non-cancer colon tissues. The integration of bio-inspired optimization algorithms with deep learning techniques not only contributes to the advancement of computer-aided diagnostic tools for colon cancer but also holds promise for enhancing the early detection and diagnosis of this disease, thereby facilitating timely intervention and improved patient prognosis. Various CNN designs, such as GoogLeNet and ResNet-50, were employed to capture features associated with colon diseases. However, inaccuracies were introduced in both feature extraction and data classification due to the abundance of features. To address this issue, feature reduction techniques were implemented using Fishier Mantis Optimizer algorithms, outperforming alternative methods such as Genetic Algorithms and simulated annealing. Encouraging results were obtained in the evaluation of diverse metrics, including sensitivity, specificity, accuracy, and F1-Score, which were found to be 94.87%, 96.19%, 97.65%, and 96.76%, respectively.

## 1. Introduction

In recent years, colon cancer has emerged as a significant cause of mortality, affecting millions of individuals worldwide [1,2,3]. Lifestyle factors, aging, and genetics are known to contribute to the development of colon cancer, with research establishing a clear link between the consumption of processed meats and alcohol and an increased risk of developing the disease [4,5]. Moreover, studies indicate a higher prevalence of this disease in developed countries, with approximately 65% of cases diagnosed in these regions [6].

However, traditional approaches to colon cancer diagnosis present significant challenges in terms of early identification and accurate diagnosis, making it a prevalent and life-threatening disease [5]. However, traditional approaches to colon cancer diagnosis present significant challenges in terms of early identification and accurate diagnosis, making it a prevalent and life-threatening disease [1,2,3]. To address these challenges and enhance the accuracy and effectiveness of colon cancer diagnosis, advanced technologies such as machine learning and deep learning have been explored in medical image analysis [7,8,9]. Artificial intelligence offers solutions to the limitations of traditional approaches by utilizing techniques such as artificial neural networks (ANNs), support vector machine (SVM), fuzzy methods, expert systems, and metaheuristic methods for disease diagnosis using medical images [10,11,12,13,14,15,16,17].

Additionally, innovative methods for diagnosing and categorizing colon cancer histopathological images are essential to improve the precision of diagnosis and ultimately improve patient outcomes. This revised introduction provides a clearer and more structured overview of the challenges in colon cancer diagnosis and the role of artificial intelligence in addressing these challenges. It eliminates repetitions and organizes the information in a more focused and coherent manner.

In general, progress in medical image analysis shows potential for transforming early detection and diagnosis of colon cancer, ultimately leading to improved outcomes for patients. Researchers are striving to optimize the diagnostic process and enhance the overall management of this life-threatening illness by leveraging state-of-the-art technologies and innovative algorithms. Given the significant global burden of colon cancer, innovative approaches are continuously sought to enhance diagnostic accuracy and treatment efficacy. This research addresses this need by focusing on the following key areas:

- introduction of an innovative method for categorizing colon cancer histopathological images without feature selection using PCA;
- utilization of an intelligent feature selection method with FMO algorithm to enhance the precision of colon cancer diagnosis;
- integration of AI, deep learning, and bio-inspired optimization algorithms for improved early detection and diagnosis of colon cancer;
- focus on streamlining the detection process, improving diagnostic accuracy, and ultimately enhancing patient outcomes;
- potential revolutionization of cancer diagnosis and treatment through cutting-edge technologies in medical imaging analysis.

The study organization provides a brief outline of the content in each section. Section 2 reviews the literature, Section 3 describes the materials and methods, Section 4 and Section 5 presents the results and discussion, and Section 6 provides the conclusion. This overview helps readers understand the structure of the study and anticipate the content of each section.

## 2. Literature Review

In recent years, colon cancer has claimed numerous lives, making it a significant concern worldwide [18]. Preventive measures, including a healthy lifestyle and regular screening, are essential for reducing the risk of colon cancer [19]. Diagnostic imaging plays a crucial role in identifying various diseases, including Alzheimer’s disease, Multiple Sclerosis, and colorectal carcinoma [20]. With its life-threatening nature, colon cancer requires early detection and accurate diagnosis for effective treatment [21].

Medical imaging modalities such as CT scans and MRI techniques aid in diagnosing colon cancer by detecting abnormal cell growth in the colon [22]. Lifestyle factors, aging, and genetics contribute to the development of colon cancer, with processed meats and alcohol consumption being associated with increased risk [23]. Screening methods like colonoscopy and histopathological screenings are vital for early detection and prevention [24]. Medical imaging, coupled with advancements in machine learning and deep learning, shows promise in improving the accuracy of colon cancer diagnosis [25].

Table 1 summarizes various research studies aimed at improving the diagnosis and prognosis of colorectal cancer through innovative methodologies such as automated algorithms, convolutional neural networks (CNNs), and integration of medical data with artificial intelligence (AI) techniques. Each study outlines its aims, advantages, disadvantages, and results, showcasing diverse approaches to addressing the challenges in colorectal cancer diagnosis and treatment.

## 3. Material and Methods

The proposed method for diagnosing both cancer and non-cancer patients is designed to integrate several steps for the analysis of histopathological images. Initially, the method involves gathering and pre-processing sample images from a dataset of colon diseases. The pre-processing step includes noise elimination, adjustment, and image quality enhancement techniques such as histogram balancing. The research employs color images with a light intensity range of 0 to 255 for each channel. The proposed methodology combines the Fishier Mantis Optimizer (FMO) algorithm with convolutional neural networks (CNNs) to enhance the accuracy and reliability of colon cancer diagnosis. This integration aims to improve model performance and interpretability by optimizing feature selection during CNN training. The method utilizes convolutional neural networks based on GoogleNet and ResNet-50 for feature extraction from histopathological images. Textural features of the images are extracted using CNN methods, and essential features for machine learning are calculated. Feature selection is represented as a binary challenge, with a feature vector of n dimensions indicating the presence of n features, where each element is either zero or one. The FMO algorithm is employed for feature selection due to its ability to simulate learning behavior in convolutional algorithms, its superior accuracy compared to other meta-heuristic optimization methods, and its capability to perform global and local searches optimally. The subsequent stage involves training the machine learning algorithm using the optimal feature vector for the classification of cancer and non-cancer histopathological images. The proposed method also includes creating an optimal feature vector and employing machine learning for dimensionality reduction and classification. The final stage involves evaluating the proposed method using testing data. The phases of the proposed method for distinguishing between cancer and non-cancer colon images include collecting histopathological samples related to colon diseases.

**Figure 1:**
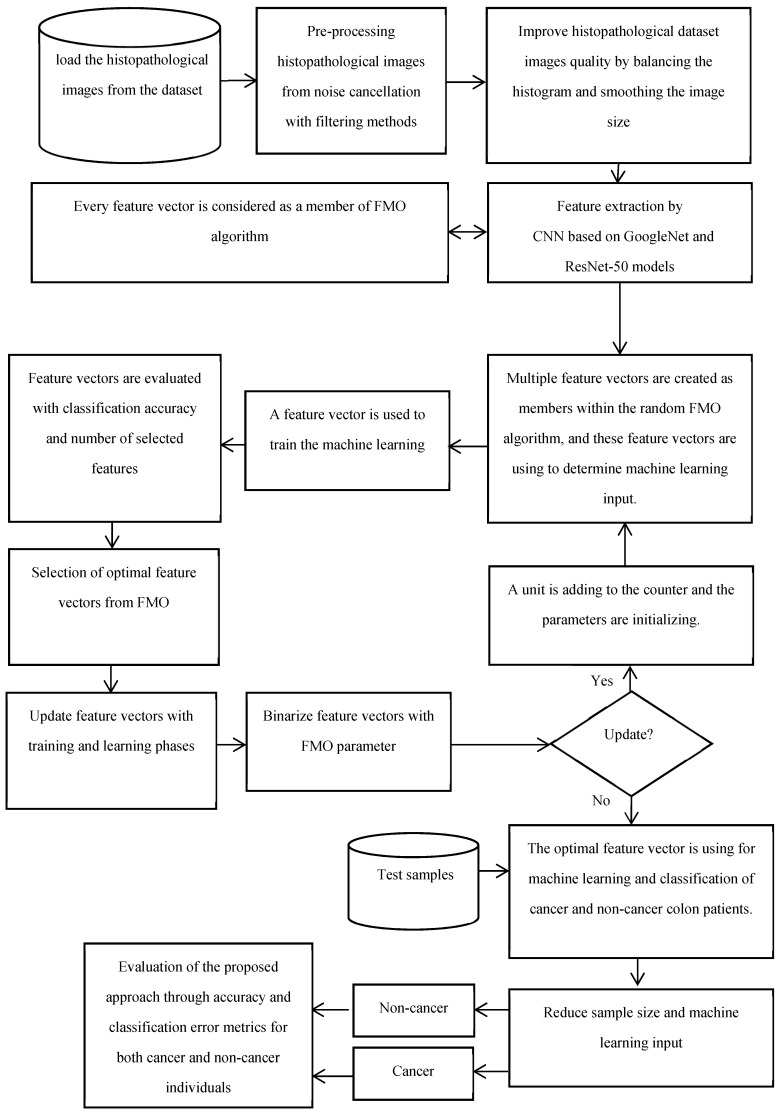
The proposed methodology’s framework for the diagnosis of patients with colon cancer.

**Figure 2:**
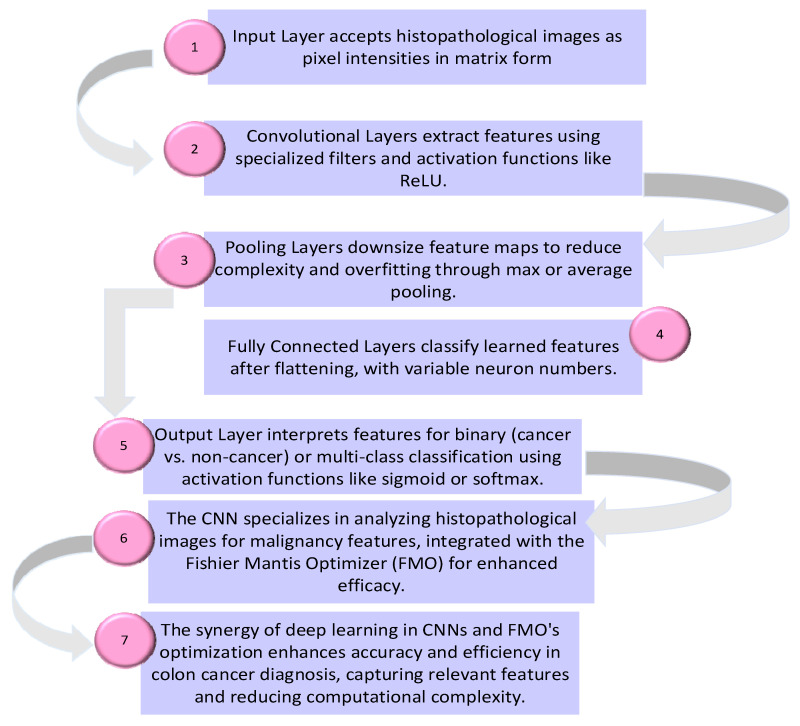
Convolutional neural network for colon cancer diagnosis.

▪ The samples are pre-processed to eliminate noise and enhance image quality.
▪ Feature extraction is performed using CNNs based on GoogleNet and ResNet-50.
▪ Textural features of the images are extracted using CNN methods.
▪ Essential features for machine learning are calculated from the images.
▪ Feature selection is represented as a binary challenge with a feature vector of n dimensions.
▪ The FMO algorithm is utilized for feature selection due to its superior accuracy and optimization capabilities.
▪ The machine learning algorithm is trained using the optimal feature vector for classification.
▪ Machine learning is employed for dimensionality reduction and classification of the images.
▪ The proposed method is evaluated using testing data to assess its performance in distinguishing between cancer and non-cancer colon images. Overall, the proposed method integrates traditional data preprocessing techniques with innovative feature selection using the FMO algorithm and CNN training to enhance the accuracy and reliability of colon cancer diagnosis. The proposed methodology’s framework for the diagnosis of patients with colon cancer is shown in Figure 1. The visual representation of the model conceptual framework in Figure 2 illustrates the seamless integration of these components, highlighting the novel approach taken in this research.

**Fishier Mantis Optimizer Algorithm**

The fisher mantis exhibits intelligent hunting behaviors, considering various scenarios and adjusting its position accordingly. It seeks the optimal location for prey or fish. Additionally, the fisher mantis displays uniform behaviors, including preparations for attacking or abandoning the current hunting state.

The proposed method makes use of the FMO algorithm outlined in reference [32]. This algorithm systematically advances through iterations, gradually bringing the mantis closer to its prey. Through this process, the algorithm progressively narrows down the potential scenarios, as illustrated in Equation (1). Here, the parameter “*m*” signifies the initial states, while “*t*” represents the states at the current iteration stage.

(1) $$m\left(t\right)=m-\frac{m\cdot it}{MaxIt}$$

The feature vector $X_{i}^{new}$ will be used in machine learning for the classification of colon images into cancerous and non-cancerous categories.

The dataset utilized in this study, “Lung and Colon Cancer Histopathological Images,” was sourced from an open-access dataset library available at “https://www.kaggle.com/datasets/andrewmvd/lung-and-colon-cancer-histopathological-images”, accessed on 10 February 2021. It comprises 25,000 histopathology images categorized into five classes. Each image is saved in JPEG format with dimensions of 768 × 768 pixels.

**Augmentation Procedure:**

To augment the dataset and increase its diversity, various augmentation techniques were applied to the original images. The augmentation process was implemented using the Augmentor package, which provides a flexible framework for image augmentation. The following augmentation techniques were utilized:

**1.**  **Rotation:**
  ▪ Range: Images were rotated within a range of angles to simulate variations in orientation.
  ▪ Angle range: [−15°, 15°].
**2.**  **Translation:**
  ▪ Shift: Images were shifted horizontally and vertically to simulate translations.
  ▪ Shift range: [−20%, 20%] of image width and height.
**3.**  **Scaling:**
  ▪ Scale factor: Images were scaled to simulate changes in size.
  ▪ Scale range: [0.8, 1.2].
**4.**  **Cropping:**
  ▪ Random cropping: Portions of the images were randomly cropped to simulate variations in composition.
  ▪ Crop size: Images were cropped to a size of 700 × 700 pixels.
**5.**  **Flipping:**
  ▪ Horizontal flipping: Images were flipped horizontally to simulate mirror reflections.
  ▪ Vertical flipping: Images were flipped vertically to introduce additional variations.

By applying these augmentation techniques with specified parameters, the dataset was augmented to a total of 25,000 images, ensuring a diverse representation of histopathological features. This augmented dataset was then used for training and evaluating the proposed method for colon cancer detection. The classification task for colon images involved distinguishing between cancerous and non-cancerous classes. The dataset consisted of 25,000 histopathology images divided into five distinct categories, with each category containing 5000 images. It effectively conveys information about the classification task and the dataset related to colon images.

**Figure 3:**
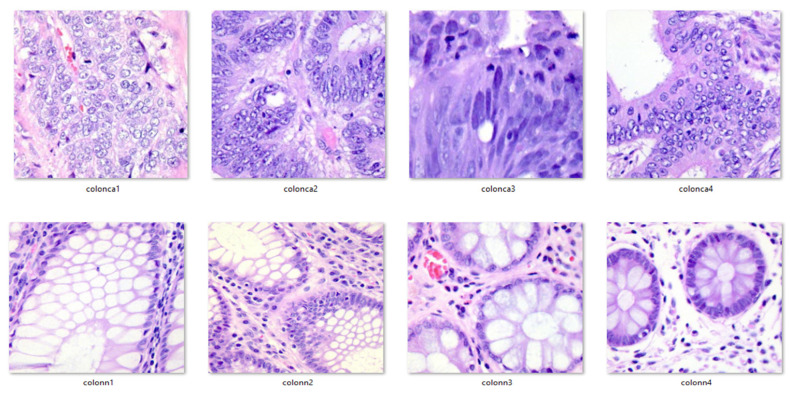
Exemplar pictures from the dataset.

Additionally, for clarity and visualization purposes, six examples of histopathological images from the dataset are provided below. Images prefixed with “colon_n_” indicate healthy colon tissue images, while those prefixed with “colon_ca_” depict images of colon cancer. Refer to Figure 3 for a visual representation of these augmented images.

Histopathological Features and Classifications:

Histopathology entails the examination of tissue samples under a microscope, often obtained through biopsies, where minuscule tissue fragments are extracted and meticulously analyzed by pathologists. This thorough examination is instrumental in identifying both cancerous and pre-cancerous cellular abnormalities. Apart from colon cancer, the colon can be affected by a range of other conditions, underscoring the importance of histopathological analysis in reaching a definitive diagnosis.

## 4. Results and Discussion

### 4.1. Classification Using Learnable Classifiers for FMO

To determine the ideal combination of techniques, a thorough investigation has been carried out. An autoencoder method and the FMO algorithm were employed collaboratively on datasets associated with colon disease to isolate and choose the most critical attributes from the input training dataset. The identical datasets used in the first model were categorized using a pre-trained CNN in conjunction with the FMO method. Some important metrics, like accuracy, F1-Score, etc., are applied to assess the effectiveness of methods created from the confusion matrix. For multiclass classification, metrics such as total accuracy, true positive rate, and false positive rate were considered. The fundamental terms used in this analysis include False Positive (FP), True Positive (TP), True Negative (TN), and False Negative (FN), which stand for positive and negative classifications, respectively.

These indicators have been used to calculate accuracy (ACC), sensitivity (True Positive Rate (TPR)), specificity (True Negative Rate (TNR)), precision (positive predictive value (PPV)), negative predictive value (NPV), and F1-Score as follows:

(2) $$Accuracy=\frac{TP+TN}{TP+TN+FP+FN}\times 10$$

(3) $$Sensitivity\left(True\;Positive\;Rate\left(TPR\right)\right)=\frac{TP}{TP+FN}\times 100$$

(4) $$Specificity\;(True\;Negative\;Rate\;(TNR))=\frac{TN}{TN+FP}\times 100\%$$

(5) $$Precision\;(positive\;predictive\;value\;(PPV))=\frac{TP}{TP+FP}\times 100\%$$

(6) $$Negative\;predictive\;value\;(NPV)=\frac{TN}{TN+FN}\times 100\%$$

(7) $$F1-score=\frac{2PPV\times TPR}{PPV+TPR}\times 100\%$$

### 4.2. Using Auto-Encoder with FMO for Colon Disease Dataset

Various scenarios were developed and assessed to validate the effectiveness of the proposed technique and compare different combinations. In the initial stage, a dataset related to colon illness was processed by the autoencoder, along with five different classifier types. The outcomes of the colon illness dataset using the autoencoder and the FMO technique are displayed in Table 2.

The most crucial factor in assessing this classification model is accuracy, which is based on the true values of the tested images that were classified. The KNN classifier achieved a higher accuracy rate of 75.35%.

### 4.3. Pre-Trained CNN with FMO for Colon Disease Dataset

**Figure 4:**
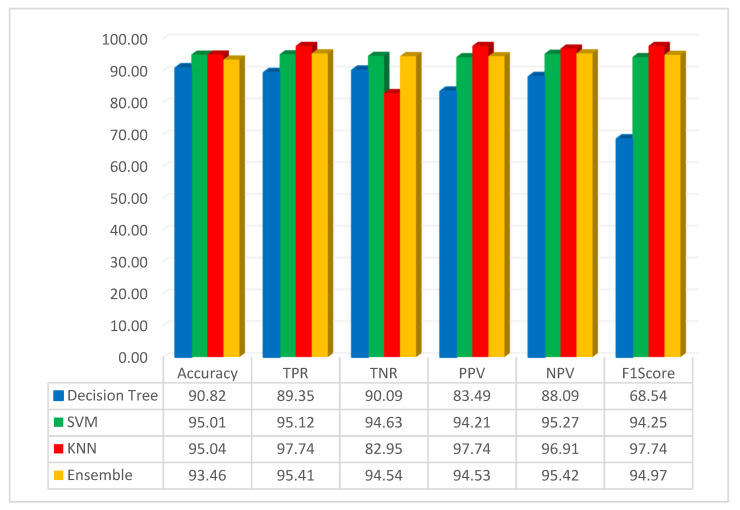
Result of the simulation based on the ResNet-50 and FMO.

The assessment of pre-trained CNN with FMO was conducted using the dataset related to colon diseases. The simulation outcome, employing the FMO method in conjunction with the pre-trained ResNet-50 network, is presented in Figure 4.

Figure 4 illustrates that the accuracy of the DT, SVM, KNN, and ensemble methods has been achieved at 90.82%, 95.01%, 95.04%, and 93.46%, respectively. The KNN classification method achieved 95.04% accuracy, which was the best result. KNN is a non-parametric supervised learning classifier that is more accurate than other methods such as SVM, decision trees, and ensemble methods. Additionally, in this scenario, the highest accuracy was achieved using the KNN classifier with features that were obtained using the FMO algorithm and a pre-trained ResNet-50 network. Additionally, this study assessed the performance of decision tree, SVM, KNN, and ensemble methods with the F1-Score metric, yielding scores of 68.54%, 94.25%, 97.74%, and 94.97% for these algorithms, respectively. As seen from the F1-Score results, it can be understood that the KNN has higher accuracy than other methods.

**Figure 5:**
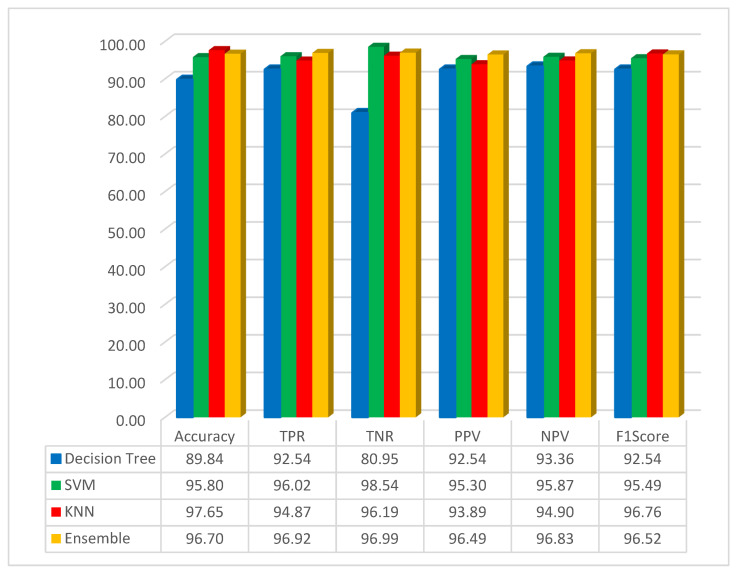
Simulation result based on the GoogLeNet with FMO.

The simulation results, based on the FMO method in conjunction with the pre-trained GoogLeNet network, are depicted in Figure 5.

As it is shown in Figure 5, the accuracy for the decision tree, SVM, KNN, and ensemble methods has been obtained as 89.84%, 95.80%, 97.65%, and 96.70%, respectively. The best result for accuracy is 97.65, obtained by the KNN classifier method. In this scenario, the KNN method has high performance compared with other methods. It can be understood that the classification with KNN and the features obtained by using the FMO and pre-trained network with the GoogLeNet has obtained the highest accuracy. In this study, the F1-Score has been implemented, and the result for the decision tree, SVM, KNN, and ensemble methods has been obtained as 92.54%, 95.49%, 96.76%, and 96.52%, respectively. As seen from the F1-Score result, it can be understood that the KNN has the highest accuracy compared to other methods.

The examination reveals that the sensitivity, specificity, and accuracy indices in the suggested approach are 94.87%, 96.19%, and 97.65%, respectively. The sensitivity index and specificity of the suggested approach outperform the methods examined in [26,33,34,35] in the examination and categorization of colon patients images, as demonstrated in Table 3.

**Figure 6:**
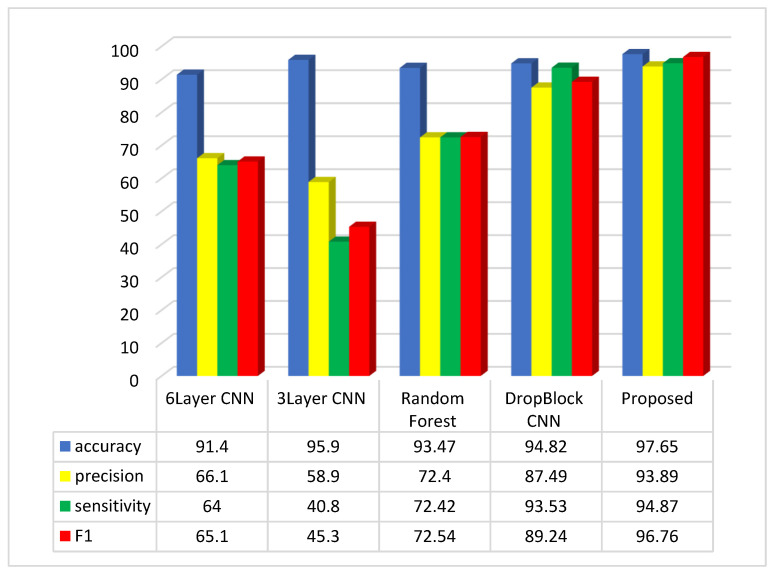
Comparative analysis of the average of accuracy, sensitivity, precision, and F1-Score in the suggested method and comparable approaches.

The accuracy score in the method outlined in [35] is recorded as 93.2%. In contrast, the suggested method attains an accuracy score of 97.65%. To conduct a comprehensive evaluation of the suggested approach, the results were compared with various investigations in the field, as illustrated in Figure 6.

The assessment suggests that the suggested approach, relying on the accuracy, precision, sensitivity, and F1-Score metrics, outperforms methods like 6Layer CNN, 3Layer CNN, Random Forest, and CNN DropBlock in colon disease image classification.

The suggested approach exhibits accuracy, precision, sensitivity, and F1-Score values of 97.65%, 93.89%, 94.87%, and 96.76%, respectively, in the colon cancer dataset image classification. Assessments indicate that the three-layer CNN method achieves the top accuracy in image categorization after the suggested approach. Regarding the accuracy metric, the CNN 6Layer approach exhibits the lowest outcome in categorization, with an accuracy of approximately 91.4%. Conversely, the proposed method attains the highest accuracy index in image classification. In terms of the sensitivity index, the suggested approach delivers the best performance, and subsequently, the CNN DropBlock approach technique had the greatest sensitivity index. The CNN 3Layer technique demonstrates the poorest sensitivity index among the compared methods. Among the compared methods, the suggested approach demonstrates the best performance in the F1 index, while the 3Layer CNN method exhibits the poorest performance in this index.

The proposed method was compared with CNN models such as ResNet and GoogleNet to show the influence of the Fishier Mantis Optimizer in the results. Also, in this paper, we used a CNN with three and six layers. These types of CNN based on the FMO give higher accuracy results than CNNs with nine and seven layers.

**Figure 7:**
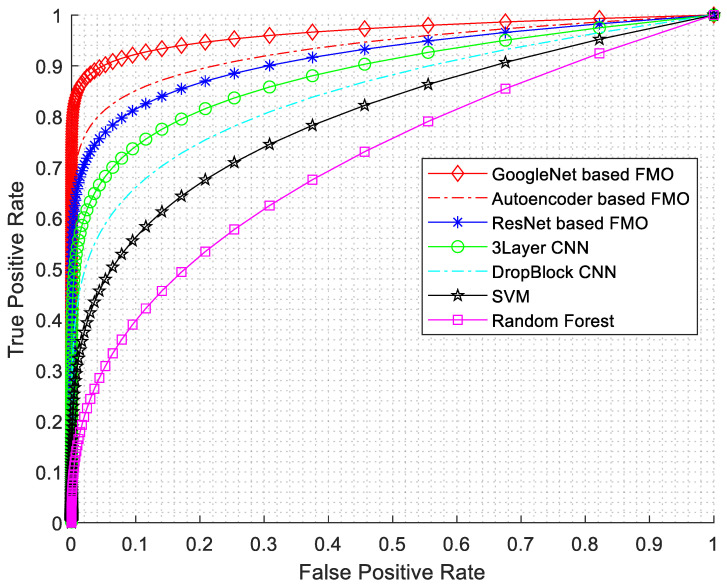
ROC diagram with AUC numerical results.

In this research, various evaluation criteria have been employed, encompassing sensitivity, specificity, accuracy, precision, F1-Score, and ROC diagrams. The ROC diagram is presented in Figure 7.

As seen from Figure 7, the best results have been obtained for GoogleNet based on the FMO algorithm. The GoogLeNet architecture consists of multiple inception modules stacked together, resulting in a network with remarkable depth and accuracy. Furthermore, the features selected by the FMO algorithm can act as auxiliary classifiers in intermediate layers, thus addressing the vanishing gradient problem.

Table 4 shows how the proposed method compares with other metaheuristic techniques like Genetic Algorithm (GA), Particle Swarm Optimization (PSO), Ant Colony Optimization (ACO), and Gray Wolf Optimization (GWO) algorithms using the same CNN based on the ResNet-50 method in terms of sensitivity, specificity, accuracy, and F1Score index findings. The following represents the mean values of the suggested method for the two classes’ sensitivity, specificity, accuracy, and F1-Score in comparison to alternative approaches.

The study’s conclusions indicate that the suggested method has an average sensitivity of 94.87%, specificity of 96.19%, accuracy of 97.65%, and F1score index of 96.76%. After comparing the proposed technique’s sensitivity, specificity, accuracy, and F1-Score to those of several existing metaheuristic methods, it was discovered that the recommended method performed better in the analysis and classification of colon patient photos. The accuracy index for the GA method comes out to be 93.56%. As part of the recommended methodology, this index is currently 97.65%. Table 4 shows that the GA algorithm produces the worst outcomes. Sensitivity, specificity, accuracy, and F1Score results for the GA algorithm were 91.15%, 94.89%, 93.56%, and 93.21%, respectively.

Early stopping was used during fine-tuning to prevent overfitting by halting training when the model’s performance on a validation set stopped improving. The best parameters were determined through a combination of grid search and cross-validation, where various hyperparameter combinations were systematically evaluated, and the set yielding the highest validation accuracy was selected.

### 4.4. The Advantages of This Study Can Be Summarized as Follows

**High accuracy:** The use of convolutional neural network (CNN) allows for the extraction of intricate features from medical images, contributing to high accuracy in colon cancer diagnosis.

The integration of the Fishier Mantis Optimizer enhances the optimization process, potentially leading to a more fine-tuned and accurate model.

**Automated Diagnosis:** The proposed approach provides an automated solution for colon cancer diagnosis, reducing dependence on manual examination and potentially speeding up the diagnostic process.

**Advanced Image Analysis:** CNNs excel in image recognition tasks, making them well suited for the analysis of medical images. This enables the model to learn complex patterns and structures indicative of colon cancer.

Nature-Inspired Optimization: The Fishier Mantis Optimizer introduces a bio-inspired optimization algorithm, potentially overcoming challenges associated with traditional optimization techniques. This can lead to improved convergence and parameter tuning.

**Timely Intervention:** The automated and accurate diagnosis facilitated by the proposed approach can contribute to timely intervention and treatment, potentially improving patient outcomes and prognosis.

### 4.5. The Disadvantages of This Study Can Be Summarized as Follows

**Data Dependency:** The success of CNNs is often dependent on large and diverse datasets. If the dataset used for training is not representative or lacks diversity, the model may not generalize well to unseen data.

**Computational Intensity:** Training deep learning models, especially CNNs, can be computationally intensive and time-consuming. This may pose challenges in terms of resource requirements, especially for institutions with limited computational capabilities.

**Complex Model Architecture:** The complexity of the CNN architecture may lead to difficulties in model interpretation and explainability. Understanding the inner workings of the model may be crucial for gaining trust in medical applications.

**Optimization Challenges:** While the Fishier Mantis Optimizer introduces a nature-inspired approach to optimization, its performance can be sensitive to the choice of hyperparameters. Finding the optimal configuration may require additional experimentation.

**Generalization Issues:** The model’s performance on new, unseen data is crucial for its real-world applicability. If the model overfits the training data, it may not perform well on diverse datasets, limiting its generalization capabilities.

In summary, while the proposed approach offers several advantages, it is essential to be aware of potential challenges and limitations, such as data dependencies, computational intensity, model complexity, and ethical considerations, to ensure the responsible development and deployment of the diagnostic.

## 5. Discussion

While this study demonstrates promising results for CNN-based colon cancer diagnosis, several avenues exist to further enhance model robustness and clinical applicability. Future work should prioritize collecting diverse and representative datasets, particularly those encompassing rare pathologies often underrepresented in training data. Furthermore, exploring advanced computational optimization techniques, such as model pruning or distributed computing frameworks, could address limitations imposed by computational intensity. Finally, rigorous validation on external datasets and in real-world clinical settings is paramount. Collaboration with clinicians and domain experts during this validation phase will be crucial for ensuring model interpretability, addressing ethical considerations, and ultimately building trust in AI-driven diagnostic tools.

## 6. Conclusions

Colon cancer poses a significant threat to public health, demanding timely and precise diagnosis for effective treatment outcomes. This study introduces a novel approach that amalgamates convolutional neural networks (CNNs) with the Fishier Mantis Optimizer (FMO) for automating the classification of colon cancer. Leveraging deep learning techniques, particularly CNNs, enables the extraction of intricate features from medical imaging data, thereby facilitating the development of a robust and efficient diagnostic model. Inspired by the hunting behavior of mantis shrimps, the FMO algorithm fine-tunes CNN parameters, enhancing model convergence speed and overall performance. This hybrid methodology aims to leverage the strengths of both deep learning and nature-inspired optimization, thereby improving the accuracy and effectiveness of colon cancer diagnosis.

Experimental validation conducted on a comprehensive dataset of colon cancer images showcases the superiority of the proposed method over traditional diagnostic approaches. The CNN-FMO model exhibits remarkable sensitivity, specificity, and overall accuracy in discriminating between cancerous and non-cancerous colon tissues. Notably, FMO-based feature selection outperforms conventional methods like Genetic Algorithms and simulated annealing, resulting in superior performance metrics including sensitivity, specificity, accuracy, and F1-Score.

Furthermore, the seamless integration of established data preprocessing techniques with FMO-based feature selection and CNN training enhances the extraction of critical features from histopathological images. This integration not only improves the model’s ability to differentiate between cancer and non-cancer samples but also enhances interpretability.

The iterative optimization of CNN weights during training with FMO contributes to a more finely tuned and accurate diagnostic model for colon cancer. Addressing challenges associated with feature abundance, the incorporation of FMO algorithms improves both model performance and interpretability.

Overall, the proposed method demonstrates significant potential for enhancing early detection and diagnosis of colon cancer, thereby facilitating timely intervention and improving patient prognosis. By combining deep learning with nature-inspired optimization, this study underscores the promise of innovative approaches in advancing healthcare outcomes.

## Figures and Tables

**Table 1:** Advancements in colorectal cancer diagnosis and prognosis: a comparative study of innovative approaches.

Aims	Advantages	Disadvantages	Results	Ref
1. Develop an automated algorithm for detecting and categorizing hyperplastic and adenomatous colorectal polyps during colonoscopy. 2. Utilize transfer learning from large nonmedical datasets to enhance the precision of polyp detection and classification. 3. Investigate the capability of the proposed method to aid endoscopists in promptly resecting adenomatous polyps.	1. The algorithm streamlines the process of identifying and categorizing colorectal polyps, thereby reducing the time and expenses associated with manual examination. 2. Demonstrating high precision, recall rate, and accuracy compared to visual inspection by endoscopists, the algorithm potentially mitigates the risk of overlooking adenomatous polyps. 3. Drawing insights from nonmedical datasets enriches the algorithm’s performance, underscoring the efficacy of transfer learning in analyzing medical images. 4. By precisely identifying adenomatous polyps, the algorithm facilitates prompt resection before they progress into invasive cancer, which could enhance patient outcomes.	1. Despite the effectiveness of transfer learning, there may be a disconnect between nonmedical source tasks and medical target tasks, necessitating further exploration to optimize feature selection. 2. Gathering and annotating a substantial volume of medical data to fine-tune CNN architecture may pose challenges, given the limited availability and complexity of medical datasets. 3. The proposed method may demand sophisticated computational resources and expertise in deep learning, potentially limiting its applicability across all healthcare settings.	The proposed algorithm demonstrated comparable precision but superior recall rate and accuracy compared to visual inspection by endoscopists. Outperforming prior state-of-the-art methods with minimal preprocessing, the algorithm proved effective in assisting endoscopists in identifying overlooked adenomatous polyps. These encouraging outcomes suggest that the proposed method holds promise for enhancing the early detection and diagnosis of colorectal cancer, ultimately leading to improved patient outcomes.	[26]
1. Develop a Dual-Path Convolutional Neural Network (DP-CNN) to automatically detect intestinal polyps from colonoscopy images. 2. Validate the effectiveness of the proposed DP-CNN model in detecting polyps through experimental results. 3. Evaluate the performance of the proposed method in terms of recall, precision, F1-Score, F2-Score, and overall accuracy across different databases.	1. The proposed DP-CNN model exhibits high recall, precision, F1-Score, and F2-Score in identifying polyps in both the CVC ColonDB and ETIS-Larib databases. 2. The proposed method offers lower complexity and fewer learnable parameters compared to existing deep learning models, making it suitable for real-time applications and scenarios with limited computing resources. 3. The DP-CNN architecture, coupled with a sigmoid classifier, effectively identifies polyps from colonoscopy images, facilitating early diagnosis and intervention.	1. The study focuses specifically on detecting polyps from colonoscopy images and may not be directly transferable to other medical image analysis tasks. 2. The performance of the proposed method is contingent on the quality and diversity of the training datasets, potentially restricting its applicability to different patient demographics or imaging conditions. 3. Further optimization and fine-tuning of hyperparameters may be necessary to enhance the robustness and generalizability of the proposed method across various datasets and clinical environments.	The DP-CNN model achieves high accuracy in detecting polyps, with recall rates of 99.20% and 92.85%, precision rates of 100% and 89.81%, F1-Scores of 99.60% and 91.00%, and F2-Scores of 99.83% and 89.91% on the CVC ColonDB and ETIS-Larib databases, respectively. Comparative analysis reveals superior performance compared to existing methods, demonstrating its potential for automating polyp detection and enhancing early colorectal cancer diagnosis.	[27]
1. To innovate ensembles integrating convolutional neural networks (CNNs) and transformers for semantic segmentation. 2. To validate the efficacy of merging diverse models, training methodologies, and optimization techniques to forge more potent ensembles. 3. To propose a novel approach for acquiring segmentation masks via intermediate prediction averaging. 4. To extend findings to diverse application domains and investigate strategies for adapting the model to resource-constrained hardware.	1. Ensembles combining CNNs and transformers exhibit superior polyp segmentation compared to alternative methods. 2. Intermediate prediction averaging diminishes overfitting, bolstering the model’s resilience. 3. The proposed methodology displays potential across multiple domains beyond polyp segmentation. 4. Ongoing research will explore distillation methods and pruning techniques to tailor the model for low-cost hardware.	1. Ensembles may necessitate substantial computational resources for both training and inference. 2. Performance could fluctuate based on dataset characteristics used for training and evaluation. 3. Ensembles pose challenges in discerning the contributions of individual models to the final segmentation outcome.	The devised ensembles excel across five major polyp segmentation datasets, notably outperforming leading methods on two datasets without specific fine-tuning. A novel strategy of averaging intermediate predictions significantly contributes to mitigating overfitting and refining model contributions, underscoring its pivotal role in the ensembles’ success	[28]
1. Create MEDomics, a dynamic infrastructure for organizing diverse health data and ensuring data quality. 2. Utilize artificial intelligence to predict individual prognosis in oncology using MEDomics. 3. Validate the effectiveness of the MEDomics framework in oncology by identifying correlations between clinical factors and mortality. 4. Utilize natural language processing (NLP) to continuously update prognosis estimates as disease conditions evolve.	1. MEDomics systematically organizes health data and continuously evaluates data quality. 2. The framework identifies clinically significant associations, such as the strong link between the Framingham risk score and cancer mortality. 3. Discoveries like the Framingham risk score’s impact on mortality can guide clinical decisions, potentially enhancing patient outcomes. 4. NLP enables ongoing adjustments to prognosis estimates, enabling personalized and timely interventions.	1. Many hospitals may lack readiness to integrate data science into clinical workflows, hindering the widespread adoption of systems like MEDomics. 2. Ensuring data accuracy and reliability within MEDomics requires ongoing attention and resource allocation. 3. The complexity of AI algorithms and NLP techniques may impede understanding among clinicians and healthcare providers. 4. Developing and sustaining dynamic infrastructures like MEDomics entails substantial investments in personnel, technology, and infrastructure.	MEDomics proves its efficacy in oncology by revealing the strong association between the Framingham risk score and cancer mortality across different stages. Integration of NLP facilitates continual prognosis updates, adapting to evolving disease conditions. This framework offers a promising avenue for leveraging AI and diverse health data to enhance individual prognosis and guide clinical decision-making in oncology.	[29]
1. Demonstrate the viability of integrating serum Raman spectroscopy with a convolutional neural network (CNN) model for the diagnosis of four cancer types: gastric, colon, rectal, and lung cancer. 2. Assess the accuracy of this integrated approach and visualize the CNN-extracted features specifically for rectal cancer diagnosis. 3. Explore the potential of using serum Raman spectroscopy and CNN to differentiate between cancer and healthy individuals, with a particular focus on rectal cancer.	1. The amalgamation of serum Raman spectroscopy and CNN achieved a notable classification accuracy of 94.5%, showcasing its effectiveness in diagnosing diverse cancer types. 2. The visualization of CNN-extracted features aids in deciphering chemical compositions, offering potential insights into cancer development mechanisms. 3. Serum Raman spectroscopy presents a cost-effective, rapid, and non-destructive method for cancer screening, potentially facilitating prompt detection and intervention.	1. The opaque nature of CNN models impedes transparency in understanding the learning process, potentially limiting interpretability in the diagnostic process. 2. Despite the high accuracy observed, the precise mechanisms underlying biochemical substances in different cancer types remain incompletely understood, necessitating further research for clarification. 3. While the study focuses on diagnosing four specific cancer types, the applicability of the approach to other cancer types may require additional validation and optimization efforts.	The integration of serum Raman spectroscopy with a CNN model achieved a notable 94.5% accuracy in diagnosing multiple cancer types. Visualization of CNN features highlighted significant differences between cancer and healthy samples, indicating potential for non-invasive cancer screening and warranting further research into its mechanisms and applicability.	[30]
1. Develop an automated system employing convolutional neural network (CNN) and Ranking algorithm for colorectal cancer detection, aiming to alleviate pathologists’ workload and enhance diagnostic accuracy. 2. Assess the feasibility and efficacy of utilizing deep learning techniques in tissue-based diagnostics, utilizing openly available digital pathology datasets. 3. Investigate the potential of integrating CNN and Long Short-Term Memory (LSTM) to optimize performance and efficacy in colorectal cancer diagnosis and potentially extend applicability to diverse cancer types.	1. The proposed model exhibits superior prediction accuracy compared to existing methods, potentially facilitating early detection and prevention of colon cancer. 2. Serving as a screening tool, the automated system has the potential to reduce pathologists’ workload and minimize diagnostic subjectivity. 3. Incorporating CNN and LSTM enhances performance and expedites diagnosis, thereby improving system efficiency.	1. Manual feature selection from datasets may be laborious and could potentially impede system efficiency, necessitating meticulous consideration and potential optimization. 2. The model’s focus on detecting colorectal cancer may constrain its utility to other cancer types, warranting further research to broaden its scope. 3. The model’s performance may be influenced by the quality and diversity of input datasets, potentially limiting its generalizability across various populations or imaging conditions.	The proposed model, employing CNN and Ranking algorithm, demonstrates superior performance in colorectal cancer diagnosis compared to existing methods, as indicated by higher Recall, Precision, and Accuracy metrics. Integration of CNN and LSTM enhances the model’s efficiency and opens avenues for potential expansion to identify various cancer types, promising advancements in medical image diagnosis frameworks.	[31]
1. Explore the spatial distribution of T cell subsets in the tumor microenvironment among colon cancer patients. 2. Establish connections between spatial T cell distribution and previously analyzed genomic data in the AC-ICAM colon cancer patient group. 3. Investigate the potential prognostic significance of T cell spatial distribution concerning patient survival and Immunologic Constant of Rejection (ICR) transcriptome correlation.	1. Provides valuable insights into the intricate interplay between immune cells and the development of colon cancer. 2. Integrates spatial T cell distribution data with genomic insights, enhancing comprehension of tumor-immune dynamics. 3. Offers potential prognostic implications by associating T cell spatial distribution with patient survival and ICR transcriptome correlation.	1. Restricted to a specific cohort of colon cancer patients (n = 90), which could limit applicability to broader patient populations. 2. Relies on a specialized multiplex immunofluorescence assay, potentially introducing technical limitations and variability. 3. Requires additional validation and replication in larger cohorts to confirm the prognostic relevance of T cell spatial distribution in colon cancer	Tumor-infiltrating T cell subtypes showed comparable densities, with proliferative and Granzyme B-expressing T cells located mainly within the tumor epithelium. Immune-active subtypes exhibited increased immune cell density and reduced distances between certain T cell subtypes and tumor cells, correlating with improved survival outcomes.	[32]

**Table 2:** Dataset on colon disease auto-encoder using FMO feature selection algorithm.

Method	ACC	TPR	TNR	PPV	NPV	F1-Scoce
Decision Tree	67.80	67.94	67.94	68.20	68.20	67.93
SVM	68.40	73.83	73.83	79.80	79.80	71.63
KNN	75.35	77.05	77.05	78.50	78.50	76.10
Ensemble	73.00	74.63	74.63	76.30	76.30	73.86
Naive Bayes	66.60	72.74	72.74	80.10	80.10	70.57

**Table 3:** Average sensitivity, specificity, and accuracy comparison between the suggested method and comparable approaches.

Reference	Sensitivity	Specificity	Accuracy
R. Zhang et al. [26]	85	87	83
Y. Shin and I. Balansingham [33]	83	84	83
W. Ryan et al. [34]	89	89	89.4
Poudel, S et al. [35]	93	92.8	93.2
**Proposed Method**	**94.87**	**96.19**	**97.65**

**Table 4:** Comparative results of GA, PSO, ACO, and FMO based on sensitivity, specificity, accuracy, and F1-Scores.

Method	Sensitivity	Specificity	Accuracy	F1Score
GA-CNN	91.15%	94.89%	93.56%	93.21%
PSO-CNN	92.12%	95.22%	95.13%	94.57%
ACO-CNN	92.35%	95.90%	95.98%	95.89%
GWO-CNN	93.23%	95.67%	96.68%	95.23%
**FMO-CNN**	**94.87%**	**96.19%**	**97.65%**	**96.76%**

## Data Availability

The data presented in this study are openly available in kaggle repository at https://doi.org/10.48550/arXiv.1912.12142, reference number [LC25000].
